# Impact of Simulated Gastrointestinal Conditions on Antiglycoxidant and α-Glucosidase Inhibition Capacities of Cyanidin-3-*O*-Glucoside

**DOI:** 10.3390/antiox10111670

**Published:** 2021-10-23

**Authors:** Didier Fraisse, Alexis Bred, Catherine Felgines, François Senejoux

**Affiliations:** Unité de Nutrition Humaine, Université Clermont-Auvergne, INRA, UNH, CRNH Auvergne, F-63000 Clermont-Ferrand, France; didier.fraisse@uca.fr (D.F.); alexis.bred@uca.fr (A.B.); catherine.felgines@uca.fr (C.F.)

**Keywords:** cyanidin-3-*O*-glucoside, kuromanin, anthocyanin, antioxidant, glycation, glucosidase, digestion, bioaccessibility

## Abstract

Cyanidin-3-*O*-glucoside (C3G) is a widespread anthocyanin derivative, which has been reported in vitro to exert potent antioxidant, antiglycation and α-glucosidase inhibition effects. Nevertheless, the physiological relevance of such properties remains uncertain considering its significant instability in gastrointestinal conditions. A simulated digestion procedure was thus instigated to assess the influence of gastric and intestinal media on its chemical integrity and biological activities. HPLC analyses of digested C3G samples confirmed the striking impact of intestinal conditions, as attested by a decomposition ratio of 70%. In contrast, with recovery rates of around 90%, antiglycation, as well as DPPH and ABTS scavenging assays, uniformly revealed a noteworthy persistence of its antiglycoxidant capacities. Remarkably, a prominent increase of its α-glucosidase inhibition activity was even observed after the intestinal phase, suggesting that classical in vitro evaluations might underestimate C3G antidiabetic potential. Consequently, the present data provide novel and specific insights on C3G’s digestive fate, suggesting that the gastrointestinal tract does not profoundly affect its positive action on oxidative and carbonyl stresses. More specifically, it also tends to support its regulating effects on postprandial hyperglycemia and its potential usefulness for diabetes management.

## 1. Introduction

With several hundreds of derivatives described so far, anthocyanins represent a major class of polyphenolic constituents [1,2]. Moreover, they are considered the most important water-soluble pigments in vascular plants [1] and are responsible for the red-blue color of a wide range of vegetables and fruits [3]. Constantly found as glycosides in nature, such compounds can be derived from six main anthocyanidin skeletons that comprise cyanidin, delphinidin, malvidin, pelargonidin, peonidin and petunidin aglycones [4]. Interestingly, cyanidin derivatives are particularly widespread in the plant kingdom and are reported to be the most abundant anthocyanin form in fruits and vegetables [5,6]. Furthermore, significant amounts of cyanidin 3-*O*-glucoside (C3G), also known as kuromanin, have been highlighted in several edible and medicinal species [7]. Indeed, with contents that can exceed 1000 mg/100 g of fresh weight (FW) [8], black elderberries can be regarded as one of the richest sources of C3G. Additionally, with values ranging from 15 to 190 mg/100 g FW, significant concentrations have also been detected in several widely consumed berries, such as blackberries, raspberries, chokeberries and different kinds of grapes [8,9,10].

Over the last decades, increasing attention has been paid to the health benefits of anthocyanin consumption. This class of constituents, as well as C3G, have been widely studied for their potent antioxidant activity and numerous investigations have demonstrated positive effects on oxidative stress [6,11,12]. More recently, substantial antiglycation potential has also been highlighted for C3G, suggesting an additional action on carbonyl stress [13,14]. Such properties might, at least in part, explain the preventive action of anthocyanins on several non-communicable diseases such as neurodegenerative, cardiovascular or metabolic disorders [15,16,17]. In particular, cyanidin glycosides have also been reported to exhibit protective and therapeutic potentials in diabetes and associated complications [18]. In addition to their antiglycoxidant properties, their potent inhibitory effects on α-glucosidase might contribute to their antidiabetic action by controlling postprandial hyperglycemia [18,19]. It must be noted, however, that the antioxidant, antiglycation and α-glucosidase inhibition capacities of C3G have essentially been demonstrated by in vitro investigations. It thus remains uncertain to what extent these properties are physiologically relevant.

Indeed, in vitro assessments of the biological activities of natural compounds and plant extracts is a first and fundamental step to endorse their potential physiological effects. However, such kinds of evaluations might not fully guarantee their actual benefits on human health since bioaccessibility, bioavailability and metabolization processes are not considered. In particular, the substantial sensitivity of anthocyanin constituents towards gastrointestinal conditions has been reported by several evaluations [20,21], highlighting the necessity of investigating the impact of the digestive tract on their biological properties. Interestingly, several in vitro models have recently been implemented to simulate the digestive environment [22]. By involving gastric and intestinal key enzymes and by adjusting factors such as pH, temperature and salt composition, these procedures are capable of mimicking crucial phases of the digestion process. Such methods have been shown to be effective for evaluating the bioaccessibility of a broad range of drugs, nutrients and phytoconstituents [22]. Additionally, these approaches have successfully highlighted the chemical susceptibility of several anthocyanin derivatives, including C3G, to gastrointestinal conditions [23,24]. However, further experimental data would be of major interest to provide a clearer understanding of the impact of digestive conditions on the structural integrity and the biological properties of that key member of the anthocyanin group.

The present investigation thus aimed at deciphering the digestive fate of C3G by using a simulated gastrointestinal tract model. Chemical and biological evaluations were performed to assess the impact of gastric and intestinal conditions on its structural integrity as well as its bioactivity. Estimations of the Total Phenolic (TPC) and Total Anthocyanin Contents (TAC) were first realized to appraise the intensity of induced degradation at each step of the digestive process. Additionally, HPLC-DAD analyses were implemented to provide more precise data on the engendered chemical degradation. Finally, spectrophotometric and fluorometric assays were achieved to validate the persistence of its radical scavenging, antiglycative and α-glucosidase inhibitory properties in digestive media.

## 2. Materials and Methods

### 2.1. Reagents

C3G was purchased from Extrasynthese (Genay, France). Ethanol, Methanol (MeOH) and acetonitrile (MeCN) were of chromatographic grade and were bought from Carlo Erba Reagents SAS (Val-de-Reuil, France). All aqueous solutions were prepared with pure water generated by a Milli-Q water (18.2 MΩ) device (Merck, Darmstadt, Germany). Phosphoric acid (85%), hydrochloric acid (HCl, 37% *w*/*w*) and sodium hydroxide (NaOH) were obtained from VWR Prolabo (Fontenay-sous-Bois, France). α-Glucosidase from *Saccharomyces cerevisiae* (Type I, lyophilized powder), *p*-Nitrophenyl-α-d-glucopyranoside (*p*-NPG), pancreatin from porcine pancreas (8 × USP specification), pepsin from porcine gastric mucosa (lyophilized powder, 3200–4500 units/mg protein), bovine serum albumin (BSA), 2,2-diphenyl-1-picrylhydrazyl (DPPH), 2,2′-azino-bis(3-ethylbenzothiazoline-6-sulfonic acid) diammonium salt (ABTS), d-ribose, Folin–Ciocalteu’s reagent, gallic acid, 6-hydroxy-2,5,7,8-tetramethylchromane-2-carboxylic acid (Trolox), sodium chloride, calcium chloride dihydrate, potassium chloride, magnesium chloride hexahydrate, potassium phosphate monobasic, sodium bicarbonate and ammonium carbonate were purchased from Sigma–Aldrich Chemical (Saint-Quentin Fallavier, France). DPPH and ABTS solutions were prepared every day and every half-day, respectively, and were stored protected from light at 4 °C.

### 2.2. In Vitro Gastrointestinal Digestion

**Figure 1:**
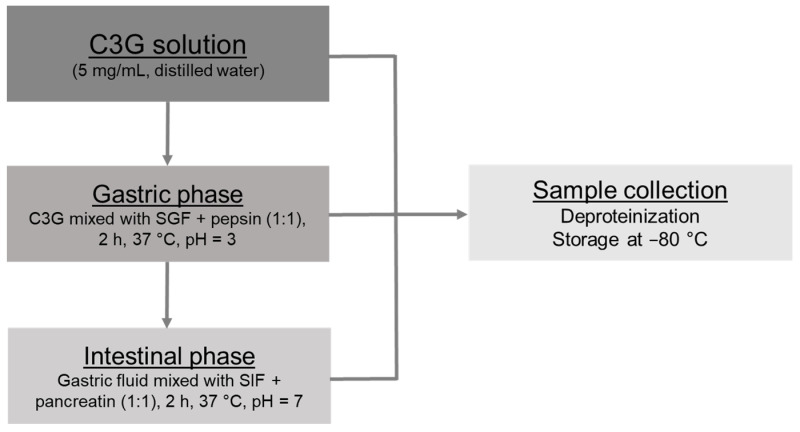
Flow diagram of the in vitro digestion protocol. C3G: Cyanidin-3-*O*-Glucoside; SGF: Simulated Gastric Fluid; SIF: Simulated Intestinal Fluid.

The in vitro digestion procedure was achieved following the standardized method published by Minekus et al. [22]. Gastric and intestinal steps were both considered (Figure 1). Simulated Gastric Fluid (SGF) and Simulated Intestinal Fluid (SIF) stock solutions were prepared in exactly the same way as Minekus et al.’s protocol [22] and similar dilutions were performed during digestion assays. All digestion experiments were realized in triplicate (*n* = 3).

#### 2.2.1. Gastric and Intestinal Phases

A simulation of gastric and intestinal phases was performed as previously reported [24]. For the gastric phase, 5 mL of C3G solution (5 mg/mL in distilled water) was mingled with 3 mL of SGF stock solution and 1 mL of pepsin solution (20,000 U/mL in SGF stock solution). A total volume of 10 mL was achieved after the addition of calcium chloride (0.075 mM in final gastric medium) and water and the pH was adjusted to 3.0 with HCl (1 M). The gastric solution was incubated for two hours at 37 °C with continuous shaking at 50 rpm in an orbital shaking incubator (NB-205 L, *N*-Biotek, Bucheon-si, Korea). The resulting gastric mixture was split in half; 5 mL was employed for the intestinal phase and 5 mL was kept back for chemical and biological assessments.

Regarding the intestinal step, 5 mL of the gastric mixture was mixed with 3 mL of the SIF stock preparation and 1 mL of the pancreatin solution (1000 U/mL in SIF solution). A total volume of 10 mL was reached after the addition of calcium chloride (0.3 mM in final intestinal medium) and water and the pH was adjusted to 7.0 with NaOH (0.1 M). An additional incubation (2 h) was completed with constant shaking (50 rpm) at 37 °C.

#### 2.2.2. Sample Management

To normalize C3G concentration in gastric and intestinal samples, a first dilution (1/2 in distilled water) was applied to the gastric solution. All samples were immediately deproteinized by adding four parts of ethanol and were subsequently centrifugated for 15 min at 4300 rpm (Centrifuge 5804 R, Eppendorf, Montesson, France). Supernatants were split in aliquots of 1 mL, which were kept at −80 °C until further experiments. A control solution of C3G was also performed (1.25 mg/mL in distilled water) to serve as a reference (undigested control). It was submitted to the same deproteinization, centrifugation and conservation processes as the digestive samples.

### 2.3. Spectrometric and Fluorometric Evaluations

The TPC and TAC were evaluated following the previously reported colorimetric methods [24]. Absorbances were measured at 740 and 520 nm, respectively, with a Jasco V-630 spectrophotometer (Lisses, France). For the TPC, a standard curve of gallic acid (5–80 μg/mL) was plotted (*R*^2^ = 0.9979, y = 4.394x + 0.021) and results were expressed as milligrams of gallic acid equivalents (mg GAE) per gram of dry sample. Regarding the TAC, a standard curve of cyanidin 3-*O*-glucoside (5–200 μg/mL) was obtained (*R*^2^ = 0.9970, y = 55.229x + 0.012) and contents were indicated as milligrams of C3G equivalent per gram of dry sample.

The DPPH scavenging activity was assessed following a previously published protocol [14]. Briefly, samples were diluted five times in water. Then, 40 µL of the solutions was mingled with 2.5 mL of the fresh radical mixture (25 μg/mL in MeOH). Incubation at room temperature was performed for 30 min and absorbance (515 nm) was monitored using a UV–vis Jasco V-630 spectrophotometer. A standard curve of Trolox (100–3000 μmol/L) was constructed (*R*^2^ = 0.9978, y = 1101.1x + 1.520) and the DPPH scavenging capacity was expressed in micromoles of Trolox equivalent (μmol TE) per gram of sample.

The ABTS scavenging capacity was evaluated as previously reported [24]. After a 10x dilution in a phosphate buffer (50 mM), 20 µL of the solutions was mixed with 250 µL of the ABTS^•+^ mixture prepared following the protocol of Re et al. [25]. A 10 min incubation was performed and absorbance was measured at 734 nm using a microplate reader (TECAN infinite F200 PRO microplate reader, Lyon, France). A standard curve of Trolox (75–300 μmol/L) was plotted (*R*^2^ = 0.9992, y = 11,760x + 0.5495) and ABTS scavenging activity was indicated in μmol TE/g of sample.

Inhibition of the Advanced Glycation End-products (AGEs) formation was carried out using the BSA/d-ribose method as previously described [26]. The AGEs fluorescence was evaluated using a microplate reader (TECAN infinite F200 PRO) with 370 and 440 nm as the excitation and emission wavelengths. Analyses were performed on five concentrations of samples (6.25–100 µg/mL). Activities were expressed as IC_50_ in µg of dry sample/mL. Recovery indexes of the antiglycation capacity were also indicated and 1/IC_50_ values of digested samples were compared to that of the undigested control.

Evaluation of α-glucosidase inhibitory activity was performed according to the previously reported method [27], with slight amendments. In 96-well plates, reaction mixtures containing 120 μL of 0.1 M phosphate buffer (pH = 6.8), 20 μL of α-glucosidase (0.25 U/mL of phosphate buffer) and 20 μL of at least five different concentrations of evaluated samples (1–100 μmol/L, final concentrations) were pre-incubated for 15 min at 37 °C. Then, 40 μL of substrate (*p*-NPG, 2.5 mM in buffer) was added and a second incubation was performed (30 min, 37 °C). Enzymatic hydrolysis led to the formation of colored *p*-nitrophenol which was recorded at 410 nm using a microplate reader (TECAN infinite F200 PRO). The control was prepared by replacing digested samples with the phosphate buffer. Acarbose was chosen as the reference α-glucosidase inhibitor and was evaluated in the same conditions at concentrations ranging from 0.01 to 1 mmol/L. Results were expressed as IC_50_ values in μmol/L.

### 2.4. HPLC Analysis

HPLC analyses were performed with a LaChrom Elite system (VWR-Hitachi, Radnor, PA, USA) consisting of two L7100 pumps, a L7200 autosampler, a L2450 diode array detector (DAD) and EZ Chrom Elite software (Agilent Technologies, Santa Clara, CA, USA). Retreated samples were diluted two times and analyzed with a reversed phase Purospher^®^ Star C_8_ endcapped column (125 × 4 mm, 5 μm particle size). A gradient elution was settled. The mobile phase was composed of water with 1% phosphoric acid (A) and MeCN (B). The gradient was set as follows: 0–5 min, 5% B; 5–30 min, 5–7% B; 30–45 min, 7–12% B; 45–50 min, 12–40% B. A flow rate of 1 mL/min, an injection volume of 20 μL and a monitoring wavelength of 520 nm were selected.

### 2.5. Statistical Analyses

The statistical significance was evaluated by one-way ANOVA, followed by a Fisher’s Least Significant Difference (LSD) test; *p* values of 0.05 or less (*p* ≤ 0.05) were considered statistically significant. All data are expressed as a mean ± the standard error of mean (SEM). All analyses were done in triplicate (*n* = 3).

## 3. Results and Discussion

### 3.1. Chemical Analyses of Digested C3G Samples

**Figure 2:**
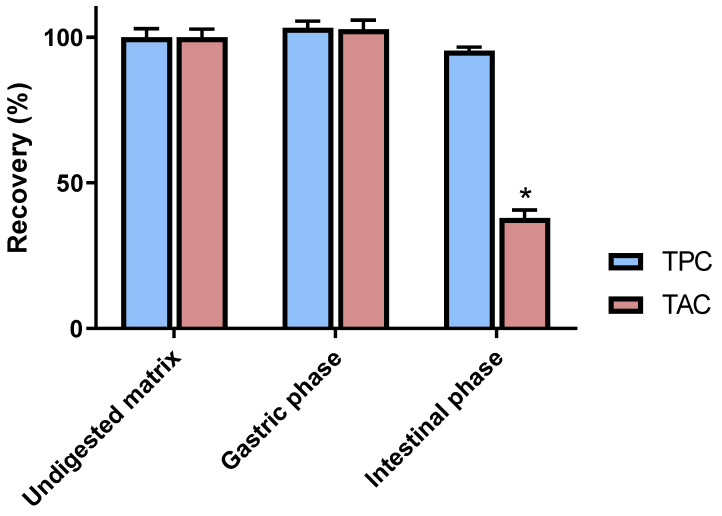
Impact of gastrointestinal simulated digestion on the Total Phenolic Content (TPC) and Total Anthocyanin Content (TAC) of cyandin-3-*O*-glucoside samples. Values are presented as means ± SEM (*n* = 3). All results are expressed as percentages, with the control (i.e., the undigested matrix) normalized as 100%. * *p* < 0.05 vs. control.

C3G and anthocyanin derivatives are reported as particularly unstable constituents and their chemical integrity can be affected by numerous factors such as pH, light or temperature [28]. Given this, the digestion process is likely to induce major structural modifications to C3G that are worthy of investigation. By using an in vitro digestion procedure, C3G was submitted to simulated gastric as well as intestinal conditions. The impacts of these treatments were first estimated by assessing the global phenolic and anthocyanin contents of generated samples. The gastric step did not induce significant modification in both anthocyanin and phenolic amounts, as attested by respective recovery rates of 102.7 ± 3.1% and 103.2 ± 2.3% (Figure 2). These data tend to indicate that C3G is not noticeably affected by simulated gastric conditions. This tendency is consistent with previous investigations on anthocyanins’ chemical behavior. Indeed, at a lower pH, such constituents have been shown to occur under a stable and red-colored flavylium form [29]. Additionally, the present results indicate that C3G is not sensitive to the gastric enzymes employed in this digestion model.

By contrast, with a quantitative recovery of 37.9 ± 2.7%, a tremendous decrease of the TAC was highlighted in an intestinal medium (*p* < 0.05). The higher pH of that second phase might be considered as an important contributing factor to C3G instability. In fact, anthocyanin pigments are known to change to a blueish-colored quinoidal form in such pH conditions [29]. Under this form, C3G has been reported to easily undergo significant transformations, leading to the polymerization and decomposition of products [30]. Interestingly, the high recovery rate of the TPC (95.4 ± 1.1%) during the intestinal phase tends to indicate that the very large majority of these degradation products are still corresponding to phenolic entities.

To provide more precise chemical data on C3G’s digestive fate, HPLC analyses were also performed on gastric and intestinal samples. These experiments were consistent with the above-mentioned TAC estimations. With a quantitative recovery of 100.7 ± 1.4%, the good stability of C3G in gastric conditions was further validated by chromatographic assays. Notably, these results are in line with previous in vitro digestion evaluations of C3G containing matrices. This constituent is actually reported to be well-preserved in a simulated gastric environment [31]. By contrast, the major influence of intestinal conditions on C3G was again observed, as attested by a prominent decrease rate of 70.0 ± 2.4%. Interestingly, these data are highly consistent with a previous in vivo study evaluating the digestive fate of anthocyanin-rich blueberries in healthy ileostomy volunteers. Collection and analyses of ileostomy effluents actually revealed an analogous reduction of 71.7% for C3G [21], ascertaining the striking impact of the digestive tract on this constituent.

### 3.2. Impact of In Vitro Digestion on Radical Scavenging and Antiglycation Activities of C3G

**Figure 3:**
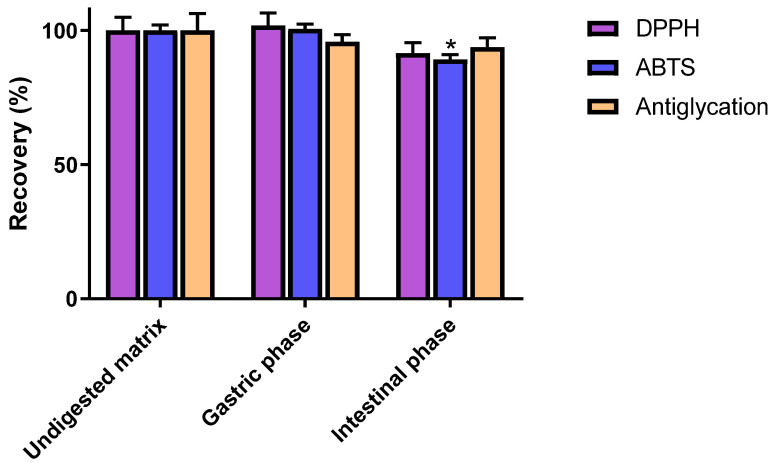
Recovery rates of radical scavenging (DPPH, ABTS) and antiglycation activities after in vitro digestion of cyanidin-3-*O*-glucoside. Data are indicated as means ± SEM (*n* = 3). All results are expressed as percentages, with the control (i.e., undigested matrix) normalized as 100%. * *p* < 0.05 vs. control (undigested matrix). DPPH: 2,2-diphenyl-1-picrylhydrazyl, ABTS: 2,2′-azino-bis 3-ethylbenzothiazoline-6-sulphonic acid.

The potent antiglycative and radical scavenging capacities of C3G have been highlighted by several in vitro evaluations [13,14]. Nevertheless, the physiological relevance of such investigations might be incomplete considering the aforementioned instability of C3G in digestive conditions. The antiglycoxidant properties of C3G were thus assessed at each step of the digestive process to provide additional information on C3G potential health benefits. Consistently with previous reports [32], prominent DPPH and ABTS scavenging properties were pointed out for undigested C3G, as is attested to by the respective values of 3761 ± 189 and 9218 ± 195 μmol TE/g (Table 1). In line with the good stability of C3G in gastric conditions, no significant modification of its antioxidant properties was observed following gastric phase. Indeed, with respective recoveries of 101.9 ± 4.7 and 100.5 ± 1.9%, its DPPH and ABTS scavenging capacities were fully preserved during that digestive step (Figure 3). That the antioxidant properties of C3G were also almost completely recovered after the intestinal phase is of major interest. Very limited reductions of around 10% were actually detected with these two assays. Notably, only the diminution of the ABTS radical scavenging capacity appeared to be statistically significant (*p* < 0.05).

Substantial antiglycation activity was also observed for undigested C3G, as was ascertained by its very low IC_50_ of 40.52 ± 2.61 µg/mL. In accordance with previous investigations [13], C3G was even shown to exhibit a stronger inhibitory effect (*p* < 0.05) than aminoguanidine (IC_50_ = 140.9 ± 5.8 µg/mL), a commonly employed positive control. As observed for radical scavenging experiments, gastric and intestinal steps did not induce major modifications of the antiglycation capacity of C3G. In fact, with respective values of 95.8 ± 2.6 and 93.8 ± 3.4%, the recovery rates of these two digestive phases were not significantly different from the undigested matrix (*p* > 0.05).

Taken together, these data indicate that antiglycoxidant properties of C3G might be slightly affected by the gastrointestinal digestion process. Nevertheless, a very good level of persistence was observed during the three performed assays. Such results clearly indicate that the intense structural modifications spotted by chemical analyses are not associated with a pronounced alteration of C3G potential benefits on radical and carbonyl stresses. It strongly suggests that C3G decomposition products also contribute to the activities observed in simulated intestinal conditions. Indeed, owing to their above-confirmed phenolic nature, these components are likely capable of exerting prominent antiglycoxidant activities.

### 3.3. Impact of In Vitro Digestion on α-Glucosidase Inhibition Properties of C3G

Located in the intestinal brush border, α-glucosidases can be regarded as key carbohydrate hydrolysis enzymes which are capable of converting non-absorbable oligosaccharides and disaccharides into absorbable monosaccharides [33]. They thus play a major role in the digestive tract by promoting glucose uptake in the small intestine, leading to an increase in blood sugar levels. Consequently, these enzymes represent a major target in the prevention and treatment of type 2 diabetes (T2D) and several inhibitors, such as acarbose, are currently employed to control postprandial glucose levels in diabetic patients [34].

**Figure 4:**
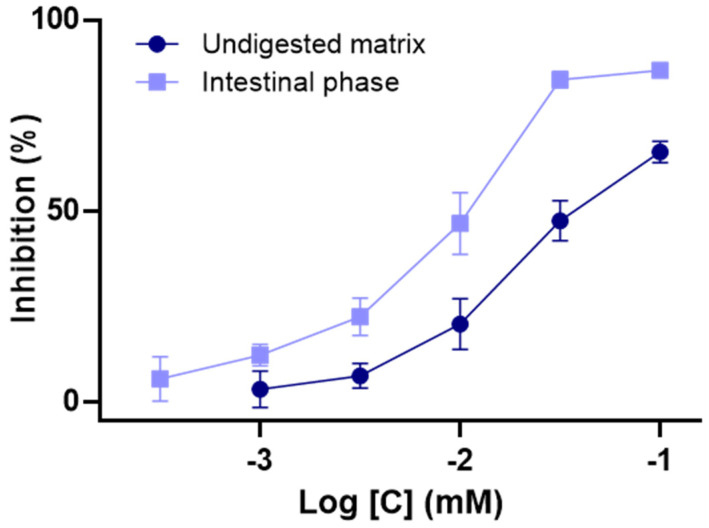
Influence of in vitro digestion on the α-glucosidase inhibition activity of cyanidin-3-*O*-glucoside. Data are indicated as means ± SEM (*n* = 3). All results are expressed as percentages.

By using a common spectrometric evaluation, α-glucosidase inhibition activities of digested samples of C3G were thus assessed to estimate its actual antidiabetic potential in physiological conditions. Considering the intestinal location of such enzymes, evaluations focused on the comparison of undigested and intestinal matrices. In accordance with previous investigations [35,36], a substantial inhibition capacity was determined for undigested C3G. Moreover, with an IC_50_ of 22.7 ± 7.1 μmol/L, C3G was shown to exert a stronger effect (*p* < 0.05) than the acarbose positive control (IC_50_ = 340.2 ± 21.2 μmol/L). As illustrated in Figure 4, the intestinal phase generated a remarkable augmentation of C3G’s inhibition activity (*p* < 0.05), as is attested to by a two-times lower IC_50_ of 10.2 ± 1.6 μmol/L. These results clearly support that an intestinal-induced chemical transformation exerts a major and positive impact on C3G’s inhibition effect. It tends also to indicate that classical in vitro evaluations of its α-glucosidase inhibitory properties might underestimate the real in vivo potential of C3G. In fact, the obtained data implies that this constituent may partially act as a prodrug whose activity gradually increases during the intestinal passage.

Interestingly, these results can be tied to previous in vivo investigations of C3G antidiabetic potential. Indeed, its marked inhibition potency on α-glucosidase should substantially contribute to its hypoglycemic effect reported in diabetic mice [36]. More generally, these data suggest that the consumption of C3G-rich fruits and vegetables might improve glycemic regulation in T2D. Notably, the reduction in fasting blood glucose, engendered by an elderberry supplementation in diabetic rats, clearly weighs in favor of such statements [37]. Moreover, the positive effects on glucose homeostasis induced by C3G-rich extracts from bayberry [38] and haskap berry [39] give further strength to this hypothesis.

## 4. Conclusions

By using a standardized in vitro digestion procedure, the present investigation ascertains the modest bioaccessibility of C3G and confirms its extensive instability in intestinal conditions. HPLC experiments actually indicated that 70% of the digested C3G suffered chemical modifications. Considering its intense digestive susceptibility, classical in vitro evaluations of C3G biological properties might not be sufficient to fully validate its health benefits. Indeed, confirmation of the persistence of its effects after gastrointestinal-induced transformations is of utmost importance. Interestingly, a remarkable preservation of its antiglycoxidant properties was determined after both the gastric and intestinal steps. In fact, with recovery rates of around 90%, antiglycation, as well as DPPH and ABTS scavenging assays, uniformly revealed a marginal impact of gastrointestinal treatment. This clearly indicates that the digestive environment does not profoundly affect the action of C3G on oxidative and carbonyl stresses and supports its potential preventive action on several non-communicable diseases. More specifically, the antidiabetic potential of C3G might even be underestimated by in vitro experiments, as is attested to by the substantial intensification of its α-glucosidase inhibition activity in a simulated intestinal medium. Consequently, the present data also suggest that the consumption of C3G-rich fruits might help to regulate postprandial glucose levels in T2D patients. Nevertheless, further evaluation of the impact of more complex food matrices on C3G’s digestive fate would be worth investigating to fit more precisely with its actual mode of consumption.

## Figures and Tables

**Table 1:** Influence of in vitro digestion on radical scavenging and antiglycation properties of cyanidin-3-*O*-glucoside.

Assay	Undigested Matrix	Gastric Phase	Intestinal Phase
DPPH scavenging activity (μmol of Trolox eq/g)	3761 ± 189 ^a^	3834 ± 176 ^a^	3445 ± 145 ^a^
ABTS scavenging activity (μmol of Trolox eq/g)	9218 ± 195 ^a^	9268 ± 172 ^a^	8225 ± 147 ^b^
Antiglycation activity (IC_50_, µg/mL)	40.52 ± 2.61 ^a^	42.00 ± 1.10 ^a^	42.97 ± 1.55 ^a^

Data are expressed as mean values ± SEM (*n* = 3). Values in the same row sharing identical superscript (a, b) are not significantly different from each other (*p* > 0.05). DPPH and ABTS radical scavenging values are indicated as milligrams of Trolox equivalent per gram. Antiglycation activity is expressed as IC_50_ in µg/mL.

## Data Availability

The data presented in this study are available in this manuscript.
